# Towards a People’s Social Epidemiology: Envisioning a More Inclusive and Equitable Future for Social Epi Research and Practice in the 21st Century

**DOI:** 10.3390/ijerph16203983

**Published:** 2019-10-18

**Authors:** Ryan Petteway, Mahasin Mujahid, Amani Allen, Rachel Morello-Frosch

**Affiliations:** 1 OHSU-PSU School of Public Health, Portland State University, Portland, OR 97201, USA; 2 School of Public Health, University of California, Berkeley, CA 94720, USA

**Keywords:** social epidemiology, social inclusion, CBPR, ICTs, neighborhoods and health, participatory research

## Abstract

Social epidemiology has made critical contributions to understanding population health. However, translation of social epidemiology science into action remains a challenge, raising concerns about the impacts of the field beyond academia. With so much focus on issues related to social position, discrimination, racism, power, and privilege, there has been surprisingly little deliberation about the extent and value of social inclusion and equity within the field itself. Indeed, the challenge of translation/action might be more readily met through re-envisioning the role of the people within the research/practice enterprise—reimagining what “social” could, or even should, mean for the future of the field. A potential path forward rests at the nexus of social epidemiology, community-based participatory research (CBPR), and information and communication technology (ICT). Here, we draw from social epidemiology, CBPR, and ICT literatures to introduce A People’s Social Epi—a multi-tiered framework for guiding social epidemiology in becoming more inclusive, equitable, and actionable for 21st century practice. In presenting this framework, we suggest the value of taking participatory, collaborative approaches anchored in CBPR and ICT principles and technological affordances—especially within the context of place-based and environmental research. We believe that such approaches present opportunities to create a social epidemiology that is of, with, and by the people—not simply about them. In this spirit, we suggest 10 ICT tools to “socialize” social epidemiology and outline 10 ways to move towards A People’s Social Epi in practice.

## 1. Introduction: Social Epidemiology and Its (Dis)Contents

*“Do epidemiologists and other public health professionals have a responsibility to ask whether the ways we think and work reflect or contribute to social inequality? Proponents of socially responsible science would answer yes. What say you?”*

[1] (p.1152)

Social equity and inclusion have become cornerstone considerations within public health research and practice, in part due to the increasing prominence of social epidemiology. In the most basic sense, the field is dedicated to the study and characterization of the social production of health and illness. From its early roots in the works of Villerme and Virchow [2], and similar work by Engels and Chadwick [2], to seminal moments like the notion of generalized susceptibility [3,4] and the distinction between causes of “cases” and causes of “incidence” [5]; to the demonstration of a “social gradient” in health across social classes [6], and the concepts of “fundamental causes” [7] and “weathering” [8], social epidemiology has grown increasingly nuanced, refined, and capable of elucidating how the social world shapes patterns of, and prospects for, health. And despite all the debates about its weaknesses and limitations, the field has left an indelible mark on how we understand and approach public health in both research and practice, adding critical empirical and theoretical contributions that have fundamentally altered how we see and study health and its determinants. Nevertheless, concerns remain about the relevance and impact of social epidemiology in current and future practice [9,10], and much reflection is warranted in regard to what social epidemiology is and what it could, or even should, be.

In one series of relatively recent articles, a group of scholars shared a discussion regarding “six paths for the future of social epidemiology” [9] to outline how social epidemiology can remain “distinct and useful” (p.844). The resultant exchanges centered mostly on methodological and mechanistic considerations. For example, emphasis was placed on improving analytical techniques and developing novel methodological approaches to better establish social mechanisms and causal pathways [9,11,12,13], as well making use of improved computational powers and data system technology (e.g., “big data”) [9,11]. Some raised caution about the overly empiricist approach currently favored within social epidemiology, noting that new/more data and complex methods will not necessarily advance the field [12]. Additional insights were offered in regard to recurring conceptual and theoretical concerns within the field, namely the need to improve work on macro-social determinants and increase our understanding of reciprocal relationships across multiple levels, to double-down on the examination of intergroup differences, and to continue pressing for sound theory to guide social epidemiology research [9,12,13]. Also of critical importance was the call for a more practicable and actionable social epidemiology research, with a key understanding that social epidemiology is a social science, and as such, is “meant to produce knowledge that can be used for social change” [12] (p.855). In this spirit, some called for use of specific and modifiable exposure levels to more clearly guide research translation into intervention possibilities [11]. Others, however, suggested taking a “realist” approach that engages larger questions capable of uncovering underlying social mechanisms, and not settling for simple associations [12]. Regardless of the route taken, as stated simply by Glymour and colleagues [11], “if we fail to translate research findings from academic journals to human health, the field is irrelevant” (p.858).

In another volume of essays, a different group of scholars weighed in with their thoughts on how to “rethink social epidemiology” [14]. The focus in this collection of essays was quite distinct from, though very much complementary to, the “six paths” collection. While some pieces extended discussion on topical methodological and conceptual concerns within social epidemiology, especially those related to place-health research [15,16,17], a noticeable and much needed amount of attention was given to deliberation over the role of values and politics in social epidemiology research, practice, and translation [18,19,20], and whether the field is generating the “right kind” of practicable and actionable evidence [14,21]. Similar to views expressed by Glymour and colleagues [11], a recurring theme in this volume was that “the products of social epidemiology must be rendered more relevant to public health and knowledge about social determinants must be put more readily into action” [21] (p.318). This theme is inextricably linked to concerns raised over values (e.g., social, political) in social epidemiology, not only because values shape social epidemiology research funding priorities, research questions, design decisions, method choices, and reporting norms (e.g., pay-for-access journals), but perhaps more importantly because values play an integral and often underappreciated role in knowledge translation for policy and social action [18,20]. As noted by some, social epidemiology in its current state, “by being overly descriptive and focused on methods, becomes almost irrelevant to policy efforts to reduce inequalities in health” [19] (p.177). If this remains the case for social epidemiology moving forward, it begs a fairly simple question—what are we really doing here?

The insights, ideas, and concerns communicated in each of these series represent very relevant and important facets for the field to engage and improve upon going forward. The exchanges certainly highlighted some key methodological, mechanistic, and conceptual challenges and promises within the social epidemiology field, and it is clear that the field is in good hands, even if just half of the ideas articulated are actively pursued over the next ten years. However, there is a notion that arguably belies all of these contributions that has garnered only minimal attention—matters of equity, inclusion, and participation of the people within the social epidemiology research/practice enterprise. Indeed, discussions regarding social epidemiology and its relevance in practice and for policy and action [11,14,21], failed to mention anything about the people whose voice and collective power is of ultimate importance in such practice, policy, and action. However, Smylie and colleagues [22] made a critical contribution in calling out the manner in which standard community health data-generating processes and data systems socially exclude community residents and routinely undermine their health and social interests. In doing so, they suggest a critical need for social epidemiologists to understand, “the connections among data, knowledge and power to ensure that data systems are reducing rather than contributing to social exclusion” (p. 70).

And it is here that we submit, perhaps more than anything else, “what’s wrong with social epidemiology” is that it is, in fact, not very social at all. With so much focus within social epidemiology on issues like social position, discrimination, racism, power, and privilege, researchers have remained curiously silent in regard to how the social epidemiology field itself is complicit in the reproduction and maintenance of related social inequity. In essence, a field dedicated to understanding the social production of health struggles to critically engage the social production of its science and the social value of its findings. And as it continues to blossom, with rare exception [22,23,24,25,26,27], very little has been said about the role of the people—study participants, so-called “N’s”, and communities in which they reside—in regard to the future of the field and its value/relevance in facilitating social action on health inequities. At some point it would seem necessary to ask whose interests is social epidemiology most immediately serving? And who is doing social epidemiology, and for whom? And how is it that the most recent reflection offered on the history, significance, and state of “social epidemiology for the 21st century” uttered not a single word in this regard [28]?

Drawing from social epidemiology, community-based participatory research (CBPR), and information and communication technology (ICT), the following sections present a vision for *A* People’s Social Epidemiology—one that is of, with, and by the people, and not simply about them. To begin, we ground this vision in ideas articulated through ecosocial theory [29]—specifically, pathways of embodiment and notions of agency, accountability, and the social production of science. We then outline the remaining core elements of A People’s Social Epidemiology as a scaffolding framework—Social Epidemiology + CBPR + ICT + Local Institutionalization (i.e., sustaining inclusive local research, practice, and training activity), suggesting place-based and environmental research as promising areas to pursue this work.

## 2. The Makings of a People’s Social Epi

### 2.1. The People + Social Epidemiology: Reconnecting with “Demos”

As social epidemiology moves further into the 21st Century, it appears that the people have a place within the field only in name, quite literally—“demos”, Greek for the people. Of course, there have been numerous critiques of social epidemiology calling for a greater emphasis on the development of sound theory for the field [10,30,31,32]. Of those advanced, none have articulated a role for the people beyond that of research subject, and only one has engaged notions of agency, accountability, and the social production of social epidemiology science: ecosocial theory [30]. Accordingly, it seems fitting that *A* People’s Social Epidemiology be rooted there.

Ecosocial theory integrates a full spectrum of processes and levels that influence health, from the sociopolitical structural forces of societies down to the physiological processes and molecular mechanisms of cells. As described by Krieger [30], the ecosocial approach “fully embraces a social production of disease perspective while aiming to bring in a comparably rich biological and ecological analysis” (p.672). Additionally, ecosocial theory situates health and its determinants within a historical, generational, and lifecourse perspective. The core constructs of ecosocial theory include (1) embodiment, (2) pathways of embodiment, (3) cumulative interplay between exposure, susceptibility, and resistance, and (4) accountability and agency. The second and fourth constructs are of particular focus here.

Pathways of embodiment involve the underlying “societal arrangements of power and property and contingent patterns of production, consumption, and reproduction” that influence health within “constraints and possibilities of our biology” [30] (p.672). These pathways, in effect, encompass the myriad ways in which social inequality, power imbalances, and resource inequities shape and constrain health opportunities. Within the context of current social epidemiology, it is clear that “arrangements” are not designed with inclusion and equity in mind, but predicated upon the assumption that social epidemiology is best done by a privileged few. The people are merely subjects, studied by credentialed outsiders possessing a power, status, and resource profile markedly different from their own. But we have somehow managed to imagine social epidemiology as being outside of social patterns of production, consumption, and reproduction, and have accordingly failed to interrogate its rather blatant inequitable state. If social processes, such as research and the discursive practice of producing public health knowledge (and the economic contingencies therein), constitute pathways of embodiment, then social epidemiology is seemingly taking a treadmill path to its future—simultaneously studying and reproducing social inequity.

Here, it is important to consider notions of agency and accountability. This construct is anchored in considerations of who is responsible for shaping and maintaining the societal arrangements of power, resources, and opportunity, and thus accountable for consequent health inequity. This construct also encourages considerations for and of all entities as actors with varying degrees of knowledge, expertise, and power whose expressions and manifestations are implicated in either the maintenance of or challenge to current conditions [29,30]. In other words, this construct challenges us to think critically about responsibility and culpability in regard to health inequities, and to assess balances of power in regard to whose voices and knowledge are valued and legitimized. Here, it is clear that current arrangements favor credentialed researchers who leverage existing positions of power and privilege to study inequities of power and privilege, the result of which is the accumulation of more power and privilege (vis a vis professional advancement, social capital gains, etc.). This select “group of experts” seeks primarily to answer research questions with potentially generalizable findings, and not necessarily to solve locally experienced and embodied social and environmental problems. Research participants tend to be viewed and valued primarily *as* data points, not as political constituents and social actors that could help explain or intervene *on* the data and its determinants. In the current form, social epidemiology does not evince any real overall commitment to acknowledging, complementing, facilitating, or enhancing the agency of the people, nor does its standard procedural array accommodate such agency, e.g., non-participatory survey-based research with subsequent (repetitive) secondary analyses of anonymized and decontextualized data. Shortcomings in this regard create a context wherein the creation of generalizable social epidemiology knowledge is deemed sufficient ends, with no requirements or guiding ethos to demonstrate that the work completed has tangibly benefited the actual people from whom the work was derived—that is, the actual samples and their communities. Under such conditions, considerations of accountability—for and within knowledge production processes and burdens/benefits therein—fall to the wayside. Appropriately realized, agency of the people could prove an invaluable asset to the field in the pursuit of population health equity.

This likely reads a bit harsh considering all that social epidemiology has done and continues to achieve. But perhaps it is time to rethink/reframe and expand what we mean by way of achievement. As Krieger notes [29], ecosocial theory “directs attention not only to the social production of disease, but also the social production of science” (p.898). Indeed, social epidemiology represents not only a process to study social inequities in health, but also an avenue to redress inequity in the production of science and knowledge. These constructs challenge us to critically appraise not only the role of the people, but also the roles and responsibilities of researchers/practitioners—as the very process of *doing* social epidemiology is an opportunity to engage questions of accountability that could benefit the field and its people alike. Social epidemiology could stand to benefit greatly by us taking a step back and interrogating its current state in this regard. What is at stake in reproducing social exclusion and inequity within the field, and how can social epidemiology practice what it preaches? Taken together, these notions, articulated through ecosocial theory, if applied to the social epidemiology field itself, offer a path forward from the social exclusion highlighted by Smylie and colleagues [22], and towards A People’s Social Epidemiology.

The growing prominence of community-based participatory research (CBPR) and the increasing utility and uptake of information and communication technologies (ICTs) afford the opportunity to move towards a more inclusive and participatory social epidemiology—an avenue to democratize (and “socialize”) social epidemiology research/practice. In short, what we have before us is an opportunity to remix and reboot social epidemiology with inclusion, equity, and action built into its fundamental operating code. It is a chance to reimagine “social”, and to revisit and recast Virchow (in a very paraphrased sense): what social epidemiology needs is full and unlimited democracy.

### 2.2. Participation + Social Epidemiology: Integrating CBPR

*“More than other subfields, social epidemiology is uniquely placed to benefit from partnerships to help generate new questions and to ensure findings are used to inform population health interventions”*.

[12] (p.855)

Participatory research approaches have had many names over the years, and their underlying assumptions and values have varied [33,34,35,36]. One of the most recognized approaches within in public health, and social epidemiology specifically [23,27], is community-based participatory research. Community-based participatory research (CBPR) has become a core element of much public health research [37,38,39]. Seen as more of an orientation to research than a method or set of methods, CBPR is generally characterized by equitable, collaborative, and mutually beneficial engagement between outside researchers, community residents, and other local stakeholders in the research process [34,39,40,41]. At its core are principles concerning equity, power, empowerment, and notions of knowledge and expertise. Specifically, CBPR differs from traditional research approaches, including those applied in most social epidemiology work to date, by (1) involving equitable participation and co-learning among study participants and academic partners, (2) building on community strengths, assets, knowledge, and expertise, (3) fostering participant empowerment and local capacity building to address the factors under study, and (4) balancing research and action.

In light of espoused social epidemiology goals, seriously entertaining CBPR notions related to equitable engagement and capacity building for action would appear not only desirable, but necessary. Engaging the people as co-learners and co-researchers, and building upon and enhancing various realms and levels of expertise and knowledge, would also appear integral given that the intention is to conduct relevant and actionable research. Policy impact, action, and social change are social epidemiology goals that require more than surveys, secondary data analysis, and publication of associations—they require working with people. As recent discussions regarding social epidemiology’s future touched upon [11,14,21], the field is essentially failing if its ever-increasing body of science/knowledge is not similarly matched by ever-increasing action on that science/knowledge. The role of social values and politics in this process should not be discounted [18,20,42,43,44,45,46,47]. We should consider ourselves fortunate that CBPR is indeed established and respected as a research orientation, as it naturally complements social epidemiology goals and can help ensure that considerations for social values and the realities of knowledge translation (e.g., for policy) are taken seriously [18].

Moreover, social epidemiology has a strong focus on social and environmental processes and exposures that are difficult to capture, measure, and act upon. Grounding social epidemiology research in CBPR affords opportunities to improve social epidemiology science in this regard [23,24,27], particularly because CBPR approaches can (1) enable the development of more refined and relevant research questions, (2) improve research design and implementation strategies, (3) improve data collection and analysis, (4) afford broader reach for dissemination, (5) provide an explicit and more direct link to knowledge translation and social action, and (6) increase local capacity to sustain research and change efforts [48,49,50,51]. The value of CBPR in addressing translational challenges, particularly in relation to these last three points, has been articulated elsewhere [39,51], with the takeaway being that CBPR is well suited to improve social epidemiology research translation for health equity.

Studies within the subfield of place-health research—most of which is concerned with social and environmental health exposures—represent perhaps the most promising and logical place to move towards this approach. Place-health research has grown rapidly over the last 20 years [52,53,54,55], with a body of work examining topics ranging from neighborhood food environments to community built and social environments, to residential segregation, to air particulates and toxic exposures. Regardless of the topic, all of this work is dedicated to examining contextual factors that communities experience and embody on a daily basis, and much of this work has focused on singular cities and/or discrete “places”, e.g., a “neighborhood” defined by census tract boundaries [52,56,57]. This makes place-health research particularly well-suited to incorporate and benefit from community-based approaches. Such integration is an opportunity to leverage the practical and procedural translational advantages of much place-based research (e.g., space-bound, locality- and/or jurisdiction-specific), while simultaneously capitalizing on the scientific and political translational advantages of harnessing place-based knowledge, insight, and expertise of the people whose lives unfold within the “place” being studied. Moreover, collaborative and participatory place-based social epidemiology, coupled with inclusive and equitable access to related data, could prove pivotal to local research translation and action efforts that frequently hinge upon local politics and agenda setting [14,21,22].

While CBPR can certainly enhance opportunities for deeper levels of community participation in social epidemiology research practice and improve prospects for translation and local action, it does not come without its own set of challenges, not the least of which are building and maintaining mutual trust and respect and managing power dynamics [34,49,58]. Other challenges relate to matters of inclusion and exclusion at the community and individual level, e.g., who can or is most likely to participate, which voices are most influential given pervading community power dynamics [36,59,60], and to social and cultural dynamics of racism, gender, and class—both between “outside” researchers and community members, and among community members [49,61,62,63]. And on a more basic level, not all research endeavors in the name of CBPR are equal, as researcher attitudes, backgrounds, and practice dispositions vary greatly, as do community histories with and capacities for research. Some scholars suggest that lack of attention to epistemological and power differences in this regard can result in a “CBPR” that, in effect, constitutes a reinscription of racism [63] and a (re)colonization of historically excluded, oppressed, and dispossessed communities [64,65]. These, among other considerations, should inform any integrative efforts of the sort we suggest here.

One promising example is the collaborative work done by Schulz and colleagues [66,67] around healthy neighborhood environments and local social determinants of health. This long-standing community-academic CBPR collaboration has actively engaged local community groups and individual residents in all aspects of the research-action continuum—from deciding what should be researched and survey instrument development, to data collection, analysis, and results dissemination. Other promising examples include the West Oakland Environmental Indicators Project (WO-EIP), a community-academic collaboration that has taken a citizen science approach to addressing environmental concerns [68,69,70], and Communities for a Better Environment’s (CBE) CBPR partnership to address cumulative impacts of environmental exposures in Richmond and Oakland, California [71,72,73]. These latter two examples speak to the value of CBPR specifically in regard to investigating local environmental toxins and air pollution, something highlighted in a recent review by Commodore and colleagues [74]. These sorts of projects/collaborations embody what notions of co-researcher relationships and co-production of scientific knowledge entail, and the explicit commitment to building local capacity and prioritizing locally experienced and actionable issues make them potential models for growing social epidemiology/CBPR collaborations. These characteristics, along with the power-sharing and transparency within the collaborations, exemplify what taking agency, accountability, and the social (co)production of social epidemiology science seriously might look like in practice.

### 2.3. Socializing Social Epidemiology: Incorporating ICTs

For social epidemiology to maximize the value and utility of CBPR, it is important to explore concrete mechanisms and tools that amplify the community and streamline the participatory in the research process. In other words, we need to explore ways to facilitate the social epidemiology/CBPR linkage to better address the challenge of inclusion, equity, and action. The rapidly developing and evolving field of information and communication technology (ICT) presents an opportunity to frame and address this challenge. ICT encompasses the development, use, and evaluation of communication devices/applications (referred to as ICTs)—such as television, smartphones, internet—that create, store, and facilitate access to and transfer of information. Existing and emergent tools, devices, and platforms offer a range of possibilities for enhancing the “social” in social epidemiology. Specifically, the rise of affordable smartphone technologies with camera and internet capabilities, the development and integration of open-source tools and interactive social media conduits, and the increasing availability of applications that lend themselves to “crowdsourcing” approaches, could potentially be harnessed as low-cost and highly-accessible avenues to facilitate critical engagement of the people and uplift community voice in social epidemiology research/practice. In other words, ICTs represent what could be a readily available way to bridge social epidemiology and CBPR processes and principles.

Perhaps the most useful and relevant conceptual and theoretical groundings for the design and use of ICTs stem largely from a subfield referred to as ICTD (or ICT4D)—information and communication technology for development. ICTD has a particular focus on the role and value of ICTs within the context of social, economic, and human development, with an eye towards facilitating equitable accessibility and benefit. While this subfield is relatively new [75,76,77], there exists a general consensus within ICTD circles that ICTs are capable of both improving and worsening prospects for human development and social equity, and that their use should accordingly be guided by considerations of ethics and equity, from design and implementation, to impact and evaluation [78,79,80].

Within the growing body of ICTD literature, there are a few lines of discourse that are particularly useful in guiding how to link social epidemiology and ICTs, and framing why such a linkage is not only timely and practical, but also intuitive both theoretically and scientifically. One line of ICT discourse of particular note here is that regarding “big” and “small” data. While recent exchanges within social epidemiology highlighted the importance of harnessing “big data” [9,11], a complementary and perhaps alternate and more suitable approach, in regard to research translation and timely action, might lie in harnessing what has been termed “small data” within ICT circles. As described by D’Ignazio and colleagues [81], Small Data is

*“a practice owned and directed by those who are contributing the data… The essence of Small Data is that such communities may not just participate in, but can actually initiate and drive such data investigations towards the better understanding of an important local issue”*.

(p.116)

They suggest, specifically in regard to investigating environmental factors, that “a bottom-up, participatory, grassroots approach to… data collection addresses the key issues of inclusion, accountability, and credibility, by building public participation into the data lifecycle” (p.116). If research data is indeed critical within our spectrum of evidence to inform policy change and social action, then the nature of a Small Data approach appears more capable of facilitating impacts on policy and social action compared to Big Data—especially if it were grounded in the principles and processes of CBPR. Much like the notion of popular epidemiology [82,83], and reflecting the broader notion of citizen science for environmental research and public health [84,85], Small Data within a CBPR orientation for the conduct of social epidemiology research could promote a level of agency, transparency, and accountability within the field that we have not witnessed to date—elements that arguably belie any genuine effort to spur meaningful social action from social epidemiology science. As suggested before, such an approach holds particular promise within place-based social epidemiology work—work that examines local social, environmental, economic, and political contexts and draws upon the people’s embodied experience of these contexts to answer research questions and, hopefully, inform local action.

Of course, ICTs, regardless of the epistemological and procedural underpinnings guiding their design and application, even if anchored within a CBPR approach, are not a panacea for all of social epidemiology’s shortcomings in regard to inclusion, equity, and participation. The challenges and pitfalls of ICTs have been discussed elsewhere [78,79,80,86], not the least of which relate to concerns over data quality, validity, and accessibility [81,87], and concerns around power, privacy, over-surveillance, and potential exploitation/co-optation [81,88,89,90]. Here, it is not hard to imagine how collaborative research using ICTs has potential to lead to a sort of extractive, (re)colonization of residents lives and experiences if not attuned to concerns of power within data collection, narrative construction, and dissemination processes, as well as to communities’ histories and experiences with/within traditional research *as* epistemic violence. For example, in the context of place-health research, questions need to be raised regarding how ICT-facilitated research—and (spatial) data narratives produced therefrom—might deepen existing spatial stigma within low-income and communities of color. And how might ICT use for research within certain communities interact with existing modes of behavioral and social surveillance as enacted through other mechanisms (e.g., community policing, public video surveillance systems)? And who “owns” the data? How might data agreements and dissemination plans reproduce power imbalances along racial, class, gender, or occupational lines (e.g., five manuscripts on the “data” and zero policy actions for the people)? Anchoring ICT use in principles/processes of CBPR can certainly help in responding to these concerns from procedural, epistemic, and distributive justice standpoints, yet these matters will ultimately rest upon the extent to which collaborative efforts remain engaged in reflexive, continuous assessment of partnership dynamics (e.g., trust) and contingencies of local context. Applications of ICTs within a social epidemiology/CBPR framework will need to be sensitive to these and other identified concerns and remain realistic about what ICTs can help achieve within given social, economic, and political contexts.

Nonetheless, within the existing mix of strengths and limitations, the use of ICTs has been common within health research and practice for some time now [91,92,93,94,95]. Indeed, an entire field, commonly referred to as mHealth, has taken off to the point of being included within the National Healthy People 2020 Goals [96]. In the most basic sense, mHealth is an approach to public health research and practice that utilizes ICTs, including smartphones, tablets, and other technological devices, tools, and platforms, e.g., social media platforms (e.g., Facebook, Twitter), crowdsourcing platforms, and collaborative communication and mapping tools, to achieve research and/or programmatic goals. Efforts have ranged from simple text message communication for medication or care management [97,98,99,100], to coordinating care systems [101,102], to disease monitoring and surveillance [87,103,104,105]. Additionally, the use of smartphones for ecological momentary assessment (EMA), GPS tracking, and web-based mapping is quickly becoming a popular approach for research examining the dynamics of built and social environments and monitoring related health behaviors and outcomes [106,107,108,109]. Stated simply, there is ample precedence and opportunity for social epidemiology to more actively and deliberately explore potential affordances of ICTs, and how such affordances can improve social epidemiology science and research translation.

Furthermore, from more of a pragmatic standpoint, prominent public health organizations have issued briefs on the need for public health to “rewire” for the future [96,110,111,112], in which recommendations were made to actively incorporate and explore the use of ICTs as part of standard practice. Other organizations have created subdivisions, programs, and/or training institutes for mHealth [113,114,115]. Additionally, there was a professional meeting of public health and medical researchers hosted by the Office of Behavioral and Social Sciences Research—“Wireless Health 2014” [116]—which focused on topics that most within social epidemiology and environmental health would consider quite “downstream” and individualistic, e.g., apps for diabetes self-management and healthcare appointment reminders. Indeed, to date, use of ICTs within public health research and practice has favored such “downstream” applications. Consequently, it has been argued that this body of work, as technologically innovative as it might be, may, in the long run, detract from and dilute efforts to more fully engage and address social determinants of health by amplifying attention on personal responsibility [88,117]. As such, now is a critical time to explore ways to incorporate ICTs within social epidemiology. Such incorporation would offer a counterbalance to current ICT use within public health, as well as present a path to popularize the field and “upgrade” it for 21st century practice.

Table 1 highlights a set of ICTs that could offer concrete avenues to more thoroughly and equitably engage research participants and their communities in the social epidemiology research/practice enterprise. Each illustrative example is included here because of a combination of its relatively low cost, collaborative usability, geolocation and social media sharing capabilities, and mixed-methods applicability—ranging from quantitative surveys to geotagged photos/videos. Examples are also included based on the authors’ experience using similar ICTs within their own research applying the People’s Social Epi framework [118,119]. Specifically, we have used smartphones and a multimedia-enabled web-based mapping platform called Local Ground [120], which shared many of the features of the platforms included in Table 1. Based on our experience using Local Ground, we believe that the platforms in Table 1 represent a solid set of tools to explore/apply the framework further. To paraphrase, “(we) would not be required to surrender rigor, but (we) would be required to share power” [25] (p.2050).

Lastly, while the ICT(D) literature suggests that the reach of mobile and internet technologies is increasingly comprehensive globally [110], existing concerns regarding the “digital divide” [121,122,123] and digital exclusion [124,125] in the context of ICTs are relevant to the framework presented here. Some of the illustrative tools/platforms we highlight in Table 1 were developed specifically for utility in developing/lower economic contexts (e.g., wherein internet connectivity is absent/unreliable, where mobile use is more common than computer use), as they retain full functionality offline and are compatible with a wide range of mobile devices. Even so, there are remaining inclusion/exclusion considerations regarding access types and uses—which, in many ways, are shaped by underlying social, economic, and cultural factors. Our goal is not to expound in any detail in this regard here, only to link social epidemiology to the broader discourse of ICT(D), highlight core connections/opportunities, and point out some duly noted concerns in the ICT field to facilitate a new realm of thinking among researchers/practitioners.

## 3. A People’s Social Epidemiology: An Introductory Framework

**Figure 1:**
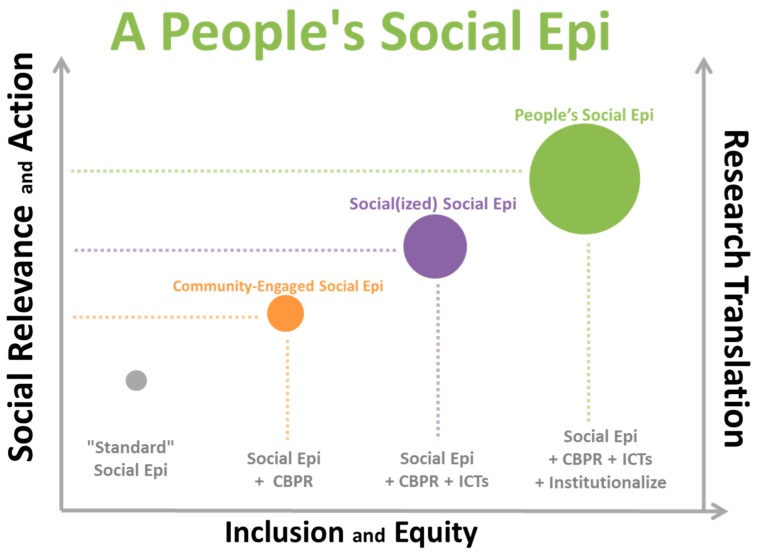
A People’s Social Epi Framework for Research Translation and Action. People’s Social Epi framework for conceptualizing how integrating social epidemiology with CBPR, ICTS, and local efforts to institutionalize this practice can improve prospects for social inclusion, equity, and action. The sizing of the circles is mainly intended to render greater visual distinction between tiers and to convey a general sense of incrementality. It is not to suggest a fixed or simple additive property of incorporating each framework component.

Figure 1 below represents a four-tiered framework for conceptualizing A People’s Social Epidemiology in regard to research translation and prospects for social action. The first tier (from left-to-right) represents what we referred to here as the “standard” social epidemiology approach—generally, social epidemiology that is non-participatory in nature, limits the role of people to being study participants, and is primarily concerned with generating science that is broadly generalizable, but not necessarily locally practicable or actionable. By preemptively excluding people from higher-level, deeper participation and devaluing their lived experience and embodied knowledge, and by not anchoring and engaging research objectives in locality-contingent social and political contexts, this standard format undermines its full potential and curtails translation and social action prospects—masking people’s agency instead of facilitating and enhancing it.

The second tier represents community-engaged social epidemiology—generally, an approach rooted in the principles and processes of CBPR. Here, the people are seen as collaborators and co-researchers, and there is an explicit focus on equitable engagement for the co-production of locally relevant and actionable science for mutual, equitable benefit. The people are simultaneously participants, scientists, collaborators, and constituents, and their voice and perspective are actively sought in all phases of the research-to-translation continuum—from defining and framing problems and deciding research questions, to collecting, analyzing, and disseminating data and determining solutions (e.g., more research, social action, policy targets). This approach to social epidemiology acknowledges that translating social epidemiology science into social action and policy change requires drawing on and building upon the knowledge and expertise of study participants and their communities and being aware of and responsive to their social and political values and those given credence within the local context where the research is being conducted. This approach to social epidemiology accordingly values participatory methodological approaches (quantitative and qualitative) that can accommodate multiple forms of knowledge expression which can be synthesized and shared via multiple formats for local consumption and impact.

The third tier represents a participatory social(ized) social epidemiology. This approach extends community-engaged social epidemiology by augmenting prospects and opportunities for the people’s participation, facilitating greater inclusion in the research-to-action process via incorporation of ICT tools and applications. Strategic use of ICTs within a community-engaged social epidemiology affords concrete mechanisms for engaging the people in the scientific enterprise—from platforms for identifying and deliberating pressing local research needs and co-developing surveys, to applications for systematically collecting, mapping, and analyzing data and organizing social action activities (Table 1). Use of ICTs within a CBPR orientation affords opportunities to not only democratize social epidemiology in research and practice, but also more readily organize, channel, and translate findings for local social action and policy debates. For example, researchers and study participants can collaboratively collect and map research data through use of web-based community mapping platforms with social media and “share” functions that facilitate easy dissemination to community, local media, and city official audiences. Participatory social epidemiology in such “social(ized)” form holds promise in uplifting and legitimizing community voice within local governance, e.g., in deliberation processes that shape social determinants of health via policy and practice. In addition to being rooted in the principles and processes of CBPR, this approach is guided by conceptual and ethical discourses regarding effective, responsible, and equitable use of ICTs. Thus, a social(ized) social epidemiology offers a conceptually rich and technology-enhanced and integrated approach to fostering inclusion and equity in social epidemiology. We have published examples of this sort of work using the People’s Social Epi framework elsewhere [118,119], making use of smartphones and a web-based community mapping platform to integrate four participatory research methodologies—all of which were executed by participants themselves.

As a final enhancement in the fourth tier, locally institutionalizing participatory social epidemiology constitutes what we call A People’s Social Epidemiology. This approach is oriented around producing practicable science and data for timely local social action and prioritizes building long-term local capacity to integrate and sustain research and social change efforts over generating diffuse and decontextualized knowledge for generalization elsewhere. A People’s Social Epidemiology proactively identifies ways to involve the people in the social epidemiology enterprise and create opportunities for their continued participation and benefit, with a belief that the people who are experiencing local social and environmental inequities are the best social epidemiologists to study and address them. Thus, a core element of this approach is creating mechanisms to not only build and sustain local capacity and legitimize local expertise, but also to facilitate more community members becoming future social epidemiology scientists—thus affecting the trajectory of not only their individual health and their communities’ health, but the health of the field. Adopting a People’s Social Epidemiology through institutionalization can of course take many forms, and the goal here is not to suggest any prescriptive extent or manner of institutionalization. However, it is worth outlining a few examples that can help illustrate the potential scope and impacts of a People’s Social Epidemiology in a local context (see Table 2 below).

## 4. Conclusions

The goal here was to outline a framework that can help social epidemiology become more inclusive and equitable. We hope that it will invigorate productive discourse regarding the field and who/what it represents in efforts to address population health inequities—such that considerations of power, representation, and procedural and epistemic justice within social and environmental research practice (e.g., who is doing this work, for whom, and how?) are more thoroughly engaged and rendered visible.

In applying this framework to our own research examining place, embodiment, and health among youth and adults [118,119], we were keen to not only take a CBPR approach that incorporated ICTs, but also to use exclusively participatory methods—training community residents to become the (paid) researchers of their own daily place-health experiences. They generated/collected, mapped, and analyzed their own data, which we then integrated for aggregate analyses. As would be expected, this work—and the trust and rapport building process behind it—took considerable time in comparison to, say, pulling up their census tract data and running secondary analyses with other administrative data. This of course has implications for consistent participation of residents within the research process, which we indeed encountered through this work (namely, adult “attrition”). From our current perspective, it is difficult to offer an overall assessment of the value or “impact” of using a People’s Social Epi approach. We believe we effectively grounded the work in ecosocial theory, and in notions and values articulated by critical and feminist theory underlying our particular CBPR orientation. Choosing methods that reflected this grounding and orientation was fairly easy, and was greatly enabled/facilitated by the use of ICTs.

With local institutionalization in mind, our project design and goals from the beginning included continuous engagement with various local/regional stakeholders. We gained the support of two city council members, the health commissioner, the city manager, the parks and recreation director, the public school superintendent, and a high school STEM director. For the latter two, we drafted plans to transform the research project into a high school STEM curriculum available to all students—with support from the health commissioner to integrate students’ “coursework” (i.e., their social(ized) social epi research) into health department community assessment practice. In this regard, considerations and prospects for mutual benefit, capacity building, and sustainability (within CBPR) were strong, as were prospects for research translation. Thus, as a general appraisal, we believe that our work sat solidly in the third tier—social(ized) social epi—and at least touched on the fourth tier.

In centering greater inclusion of the people—everyday community residents and social epi “N’s”—this framework was conceptualized at the scale at which researchers, universities, and community residents typically interact under the auspices of research—within local contexts. As we note above, we see the most direct potential for this framework in the realm of place-health research—wherein CBPR collaborations are best suited to enhance inclusion opportunities, facilitate community resident agency, and improve prospects for timely, tangible social action/research translation. We do not discount, however, the reality that macro-level political and social determinants certainly impinge upon the local—ultimately constraining the scope, impact, and feasibility of actions accordingly [126]. As articulated by Bambra and colleagues in this regard [126], it is increasingly important to envision ways to “scale up” our efforts by taking a “geographically-nuanced political economy perspective” (p.40) that engages both vertical and horizontal policy levers. As such, we would be remiss in not encouraging more explicit articulations of commitments to advancing social justice and health equity within the field, with attendant courage within policy and political realms to address macro political and social determinants. We believe that a People’s Social Epi can inform this work at/between broader levels, while recognizing that the framework’s productive value may in fact be contingent upon such work.

Our aim, it should be noted, is not to necessarily “rank” social epidemiology research endeavors going forward (at least not in any absolute, judgmental sense). It is, however, to get us wondering what criteria might be considered valuable—and priority—if such an undertaking were to unfold. It is our position here that certain criteria warrant more weight than what has been—and is presently being—afforded. The overarching premise is that greater inclusion of the people within the field can improve prospects for research translation and timely, meaningful, and (locally) relevant social action, as well as ensure that the outputs and benefits of research do not continue to disproportionately accumulate among researchers. Reappraising the value of peoples’ lived and embodied knowledge of their social and environmental contexts and experiences of social and environmental inequity, and reassessing our assumptions about the ways and degrees to which people can contribute to social epidemiology research/practice, will allow for re-envisioning how social epidemiology can make more direct and tangible impacts on the social and environmental conditions that shape health. Integrating social epidemiology with core principals and processes of CBPR and further integrating the technical and procedural affordances and theoretical groundings of ICTs can facilitate the development of a social epidemiology that is no longer simply about the people, but with and by them as well. This is how we can enhance the field and ensure it remains distinct and useful for researchers/practitioners, and more importantly, for participant communities—the “N’s” whose experiences within the social production of health are the lifeblood of our field.

## Figures and Tables

**Table 1:** 10 Potential information and communication technologies (ICTs) to “Socialize” Social Epidemiology.

	ICT Name	Features
1	Magpi	Survey instrument design, data collection & analysis; real-time assessment; SMS, photo, and audio capabilities; geolocation and mapping capability; smartphone deployment; built-in data analysis & visualization tools; collaborative use options; online/offline use
2	Fulcrum	Survey instrument design, data collection & analysis; real-time assessment; photo capability; geolocation and mapping capability; smartphone deployment; built-in data analysis & visualization tools; collaborative use options; online/offline use
3	Kobo ToolBox	Survey instrument design, data collection & analysis; real-time assessment; geolocation and mapping capability; smartphone deployment; built-in data analysis & visualization tools; online/offline use; open source
4	EthnoCorder	Survey instrument design, data collection & analysis; real-time assessment; Text, photo, video, and audio capabilities; geolocation and mapping capability; smartphone deployment; built-in data analysis & visualization tools; collaborative use options
5	MyInsights (MyPanel)	Qualitative and mixed-methods research platform for survey design, data collection and analysis; real-time assessment; Text, photo, video, and audio capabilities; geolocation capability; smartphone deployment; built-in data analysis & visualization tools; collaborative use options
6	QuickTapSurvey	Survey instrument design, data collection & analysis; real-time assessment; text and photo capabilities; geolocation capability; smartphone deployment; built-in data visualization tools; collaborative use options; online/offline use
7	FieldNotes	GPS location-based note taking and data collection & platform; real-time assessment; Text, photo, video, and audio capabilities; geolocation capability; smartphone deployment
8	LiveTrekker	GPS location-based tool for documenting geographic travel and spatial movement patterns; real-time assessment; Text, photo, video, and audio capabilities; geolocation and mapping capability; smartphone deployment; built-in data visualization tools; social media sharing tools
9	Capture365 Journal	Multimedia-enabled journaling platform; Text, photo, video, and audio capabilities; geolocation and weather tracking capability; smartphone deployment; built-in data visualization and social media sharing tools
10	MapYourWorld	Suite of mapping-focused tools geared towards youth participatory research; geolocation and mapping capabilities; built-in data visualization tools; built-in social media sharing tools

Table highlighting a selection of ICTs with the potential to facilitate “socializing” social epidemiology research practice by enabling deeply participatory and collaborative data collection, analysis, and sharing processes.

**Table 2:** 10 ways to move towards a people’s social epi through local institutionalization.

	Activity Description	Core Local Collaborators	Objectives
1	Use local social epi research study data in local health department (LHD) and city planning practice Develop data sharing/user agreements to promote open access, public dissemination	University Researchers; Health and Planning Agencies; Other social determinants of health (SDH)-related Agencies; Community Organizations	Facilitate research translation and action based on local research; Facilitate collaboration between researchers and local agencies; Promote social value and relevance of social epidemiology
2	Create Social Epidemiology/Health Equity programs within LHDs where social epi research projects are being conducted University researchers provide staff training, skill/knowledge transfer opportunities	University Researchers; LHDs	Increase LHD capacity to do social epidemiology; Facilitate collaboration between researchers and LHDs; Create opportunities for collaborative grant writing for social epidemiology research and translation activities
3	Develop local social epi Research & Practice Training Institutes Co-led by university researchers, practitioners, and residents; anchor point within LHD Create standing, rotating “Community Social Epi Fellow” position within LHD Create community-generated data program within LHD, with community social epi data teams/hubs anchored in various neighborhoods	University Researchers; LHDs; Community Organizations	Increase LHD capacity to do social epidemiology; Facilitate collaboration between researchers and LHDs; Promote social value and relevance of social epidemiology; Promote broader community understanding and knowledge of social epidemiology
4	Develop social epi “exchange program” for faculty/researchers of local universities conducting social epi research to give guest lectures at local high schools	University Researchers; High Schools	Promote social value and relevance of social epidemiology; Encourage pursuit of future public health education opportunities; Promote meaningful opportunities for researchers to connect/contribute to local communities beyond research
5	Support opportunities for local high school students to openly and freely attend courses taught by social epi researchers who are conducting research in the local community	University Researchers; Universities; High Schools	Promote social value and relevance of social epidemiology; Encourage pursuit of future public health education opportunities; Promote meaningful opportunities for researchers to connect/contribute to local communities beyond research
6	Co-Develop social epi and health equity-oriented school curricula for local high schools STEM (science, technology, engineering and math) courses exploring math, science, geography, social studies, and technology via social epi research, social epi theory, CBPR, ICTD theory, and ICT design Implement student-led social epi projects with mentorship/guidance from university researchers, graduate students Support student development of abstracts/manuscripts for professional presentation, dissemination Develop memorandum of understanding (MOU) for incorporation of student research findings within standard local health/planning practice Create standing “Youth Social Epi Fellow” position at LHD and/or planning agency	University Researchers; High Schools; LHDs	Promote social value and relevance of social epidemiology; Encourage pursuit of future public health education opportunities; Provide unique education, training, and professional development opportunities for students; Promote student connectivity to local health equity issues and facilitate their development as local change agents and future scientists
7	Develop social epi/public health college pipeline programs and/or summer institutes for local high school students Link pipeline to local community colleges, universities involved in local public health research Support student campus visits; host recruitment activities at local high schools Develop MOUs to formally support recruitment of students from communities that are current or common social epi research sites	Universities; High Schools	Promote social value and relevance of social epidemiology; Encourage pursuit of future public health education opportunities; Promote meaningful opportunities for researchers to connect/contribute to local communities beyond research
8	Create local media linkages for regular reporting/distribution of info/results/knowledge based on local social epi research projects Highlight work of local university researchers and residents currently engaged in social epi projects Develop community-written/oriented social epi journal (e.g., free, high-school reading level) focused on implications of local projects and action potential	University Researchers; Media Outlets	Promote social value and relevance of social epidemiology; Facilitate research translation and action based on local research; Promote meaningful opportunities for researchers to connect/contribute to local communities beyond research; Promote broader community understanding and knowledge of social epidemiology
9	Develop collaborations with local artists to creatively frame, represent/re-present, and disseminate social epi research findings Develop MOUs with local arts colleges to engage faculty and students as potential grant collaborators Develop standing community arts spaces to host exhibits/events to highlight research art products	University Researchers; Arts Colleges; Artists and Art Groups	Promote social value and relevance of social epidemiology; Facilitate research translation and action based on local research; Promote meaningful opportunities for researchers to connect/contribute to local communities beyond research; Promote broader community understanding and knowledge of social epidemiology
10	Support formation of standing Social Determinants Assessment and Action bodies within local government Use historic and current local social epi research data to highlight local SDH action needs across sectors/agencies Develop formal collaborations between local social epi researchers, social epi practitioners, and practitioners within local planning, housing, transportation, education, and recreation agencies and community organizations Connect collaborative work to local high school curricula to actively include youth perspectives and provide them an opportunity to shape local SDH	University Researchers; Health, Planning, Housing, Transportation, Education, and Recreation Agencies; Community Organizations; High Schools	Facilitate research translation and action based on local research; Facilitate collaboration between researchers and local agencies; Promote social value and relevance of social epidemiology

Table outlining suggestions for adopting and sustaining *community-engaged* and *social(ized) social epi* efforts within local/regional settings—standardizing/normalizing such practice to support a People’s Social Epi.

## Declaration

A version of this paper was previously published as part of one of the authors’ (R.P.) doctoral dissertation: “Towards a People’s Social Epidemiology: An Intergenerational Study of Place, Embodiment, and Health via Participatory Action Research with Residents of Public Housing,” UC Berkeley, 2016.

## References

[1] Krieger N. (1999). Questioning epidemiology: Objectivity, advocacy, and socially responsible science. Am. J. Public Health.

[2] Krieger N. (2011). Epidemiology emerges: Early theories and debating determinants of disease distribution—Poison, filth, class, & race 1600–1900. Epidemiology and the People’s Health: Theory and Context.

[3] Cassel J. (1976). The contribution of the social environment to host resistance. Am. J. Epidemiol..

[4] Syme S.L., Berkman L. (1976). Social class, susceptibility, and sickness. Am. J. Epidemiol..

[5] Rose G. (2001). Sick individuals and sick populations. Int. J. Epidemiol..

[6] Marmot M.G., Shipley M.J., Rose G. (1984). Inequalities in death—Specific explanations of a general pattern?. Lancet.

[7] Link B.G., Phelan J. (1995). Social conditions as fundamental causes of disease. J. Health Soc. Behav..

[8] Geronimus A.T. (1992). The weathering hypothesis and the health of African-American women and infants: Evidence and speculations. Ethn. Dis..

[9] Galea S., Link B.G. (2013). Six paths for the future of social epidemiology. Am. J. Epidemiol..

[10] Kaplan G.A. (2004). What’s wrong with social epidemiology, and how can we make it better?. Epidemiol. Rev..

[11] Glymour M.M., Osypuk T.L., Rehkopf D.H. (2013). Invited commentary: Off-roading with social epidemiology—Exploration, causation, translation. Am. J. Epidemiol..

[12] Muntaner C. (2013). Invited commentary: On the future of social epidemiology—A case for scientific realism. Am. J. Epidemiol..

[13] Oakes J.M. (2013). Invited commentary: Paths and pathologies of social epidemiology. Am. J. Epidemiol..

[14] O’Campo P., Dunn J.R., O’Campo P., Dunn J.R. (2012). Introduction. Rethinking Social Epidemiology.

[15] Shankardass K., O’Campo P., Dunn J.R. (2012). Place-based stress and chronic disease: A systems view of environmental determinants. Rethinking Social Epidemiology.

[16] Shankardass K., Dunn J.R., O’Campo P., Dunn J.R. (2012). How goes the neighbourhood? Rethinking neighbourhoods and health research in social epidemiology. Rethinking Social Epidemiology.

[17] Yen I.H., Shim J.K., Martínez A.D., O’Campo P., Dunn J.R. (2012). Application of two schools of social theory to neighbourhood, place and health research. Rethinking Social Epidemiology.

[18] Bayoumi A.M., Guta A., O’Campo P., Dunn J.R. (2012). Values and social epidemiologic research. Rethinking Social Epidemiology.

[19] Muntaner C., Borrell C., Ng E., Chung H., Espelt A., Rodriguez-Sanz M., Benach J., O’Campo P., O’Campo P., Dunn J.R. (2012). Locating politics in social epidemiology. Rethinking Social Epidemiology.

[20] Murphy K., Fafard P., O’Campo P., Dunn J.R. (2012). Knowledge translation and social epidemiology: Taking power, politics and values seriously. Rethinking Social Epidemiology.

[21] Mowat D., Chambers C., O’Campo P., Dunn J.R. (2012). Producing more relevant evidence: App.lying a social epidemiology research agenda to public health practice. Rethinking Social Epidemiology.

[22] Smylie J., Lofters A., Firestone M., O’Campo P., O’Campo P., Dunn J.R. (2012). Population-based data and community empowerment. Rethinking Social Epidemiology.

[23] Lantz P., Israel B.A., Schulz A.J., Reyes A.G., Oakes J.M., Kaufman J. (2006). Community-based participatory research: Rationale and relevance for social epidemiology. Methods in Social Epidemiology.

[24] Leung M.W. (2004). Community based participatory research: A promising approach for increasing epidemiology’s relevance in the 21st century. Int. J. Epidemiol..

[25] Schwab M., Syme S.L. (1997). On paradigms, community participation, and the future of public health. Am. J. Public Health.

[26] Syme S.L. (2004). Social determinants of health: The community as an empowered partner. Prev. Chron. Dis..

[27] Wallerstein N.B., Yen I.H., Syme S.L. (2011). Integration of social epidemiology and community-engaged interventions to improve health equity. Am. J. Public Health.

[28] Kawachi I., Subramanian S.V. (2018). Social epidemiology for the 21st century. Soc. Sci. Med..

[29] Krieger N. (1994). Epidemiology and the web of causation: Has anyone seen the spider?. Soc. Sci. Med..

[30] Krieger N. (2001). Theories for social epidemiology in the 21st century: An ecosocial perspective. Int. J. Epidemiol..

[31] McMichael A.J. (1999). Prisoners of the proximate: Loosening the constraints on epidemiology in an age of change. Am. J. Epidemiol..

[32] Muntaner C. (1999). Invited commentary: Social mechanisms, race, and social epidemiology. Am. J. Epidemiol..

[33] Israel B.A., Schurman S.J., Hugentobler M.K. (1992). Conducting action research: Relationships between organization members and researchers. J. Appl. Behav. Sci..

[34] Israel B.A., Schulz A.J., Parker E.A., Becker A.B. (1998). Review of community-based research: Assessing partnership approaches to improve public health. Annu. Rev. Public Health.

[35] Minkler M. (2000). Using participatory action research to build healthy communities. Public Health Rep..

[36] Wallerstein N., Duran B., Wallerstein N., Duran B., Oetzel J., Minkler M. (2017). The theoretical, historical, and practice roots of CBPR. Community-Based Participatory Research for Health: Advancing Social and Health Equity.

[37] Cashman S.B., Adeky S., Allen A.J., Corburn J., Israel B.A., Montaño J., Rafelito A., Rhodes S.D., Swanston S., Wallerstein N. (2008). The power and the promise: Working with communities to analyze data, interpret findings, and get to outcomes. Am. J. Public Health.

[38] Mercer S., Green L., Minkler M., Wallerstein N. (2008). Federal funding and support for participatory research in public health and health care. Community Based Participatory Research for Health: Process to Outcomes.

[39] Wallerstein N., Duran B. (2010). Community-based participatory research contributions to intervention research: The intersection of science and practice to improve health equity. Am. J. Public Health.

[40] Israel B.A., Coombe C.M., Cheezum R.R., Schulz A.J., McGranaghan R.J., Lichtenstein R., Reyes A.G., Clement J., Burris A. (2010). Community-based participatory research: A capacity-building approach for policy advocacy aimed at eliminating health disparities. Am. J. Public Health.

[41] Minkler M. (2010). Linking science and policy through community-based participatory Research to study and address health disparities. Am. J. Public Health.

[42] Atwood K., Colditz G.A., Kawachi I. (1997). From public health science to prevention policy: Placing science in its social and political contexts. Am. J. Public Health.

[43] Liverani M., Hawkins B., Parkhurst J.O. (2013). Political and institutional influences on the use of evidence in public health policy. A systematic review. PLoS ONE.

[44] Morgan-Trimmer S. (2014). Policy is political; our ideas about knowledge translation must be too. J. Epidemiol. Commun. Health.

[45] Muntaner C., Chung H., Murphy K., Ng E. (2012). Barriers to knowledge production, knowledge translation, and urban health policy change: Ideological, economic, and political considerations. J. Urban Health.

[46] Oliver T.R. (2006). The politics of public health policy. Annu. Rev. Public Health.

[47] Smith K.E. (2014). The politics of ideas: The complex interplay of health inequalities research and policy. Sci. Public Policy.

[48] Balazs C.L., Morello-Frosch R. (2013). The three rs: How community-based participatory research strengthens the rigor, relevance, and reach of science. Environ. Justice.

[49] Cargo M., Mercer S.L. (2008). The value and challenges of participatory research: Strengthening its practice. Annu. Rev. Public Health.

[50] Horowitz C.R., Robinson M., Seifer S. (2009). Community-based participatory research from the margin to the mainstream: Are researchers prepared?. Circulation.

[51] Cook W.K. (2008). Integrating research and action: A Systematic review of community-based participatory research to address health disparities in environmental and occupational health in the USA. J. Epidemiol. Commun. Health.

[52] Diez Roux A.V., Mair C. (2010). Neighborhoods and health: Neighborhoods and health. Ann. N. Y. Acad. Sci..

[53] Ellen I.G., Mijanovich T., Dillman K.-N. (2001). Neighborhood effects on health: Exploring the links and assessing the evidence. J. Urban Aff..

[54] Pickett K.E., Pearl M. (2001). Multilevel analyses of neighbourhood socioeconomic context and health Outcomes: A critical review. J. Epidemiol. Commun. Health.

[55] Arcaya M.C., Tucker-Seeley R.D., Kim R., Schnake-Mahl A., So M., Subramanian S.V. (2016). Research on neighborhood effects on health in the United States: A systematic review of study characteristics. Soc. Sci. Med..

[56] Chaix B., Merlo J., Evans D., Leal C., Havard S. (2009). Neighbourhoods in eco-epidemiologic research: Delimiting personal exposure areas. A response to Riva, Gauvin, Apparicio and Brodeur. Soc. Sci. Med..

[57] Leal C., Chaix B. (2011). The influence of geographic life environments on cardiometabolic risk factors: A systematic review, a methodological assessment and a research agenda: Geographic life environments and cardiometabolic risk factors. Obes. Rev..

[58] Wilson E., Kenny A., Dickson-Swift V. (2018). Ethical challenges in community-based participatory research: A scoping review. Qual. Health Res..

[59] Stoecker R. (2009). Are we talking the walk of community-based research?. Action Res..

[60] Minkler M. (2005). Community-based research partnerships: Challenges and opportunities. J. Urban Health Bull. N. Y. Acad. Med..

[61] Minkler M. (2004). Ethical challenges for the “Outside” researcher in community-based participatory research. Health Educ. Behav..

[62] Chávez V., Duran B., Baker Q.E., Avila M.M., Wallerstein N., Minkler M., Wallerstein N. (2008). The dance of race and privilege in cbpr. Community-Based Participatory Research for Health: From Process to Outcomes.

[63] Muhammad M., Garzón C., Reyes A., Wallerstein N., Duran B., Oetzel J., Minkler M., West Oakland Environmental Indicators Project (2017). Understanding Contemporary Racism, Power, and Privilege and Their Impacts on CBPR. Community-Based Participatory Research for Health: Advancing Social and Health Equity.

[64] Stanton C.R. (2014). Crossing methodological borders: Decolonizing community-based participatory research. Qual. Inq..

[65] Janes J.E. (2016). Democratic encounters? Epistemic privilege, power, and community-based participatory action research. Action Res..

[66] Schulz A.J., Kannan S., Dvonch J.T., Israel B.A., Allen A., James S.A., House J.S., Lepkowski J. (2005). Social and physical environments and disparities in risk for cardiovascular disease: The healthy environments partnership conceptual model. Environ. Health Perspect..

[67] Schultz A., Zenk S., Kannan S., Koch M., Israel B., Stokes C., Israel B., Parker E.A., Eng E., Schulz A. (2005). Community-based participatory approach to survey design and implementation: The healthy environments community survey. Methods for Conducting Community-Based Participatory Research for Health.

[68] Palaniappan M. (2004). Ditching diesel: Community-driven research reduces pollution in West Oakland. Race Poverty Env..

[69] Gonzalez P.A., Minkler M., Garcia A.P., Gordon M., Garzón C., Palaniappan M., Prakash S., Beveridge B. (2011). Community-based participatory research and policy advocacy to reduce diesel exposure in West Oakland, California. Am. J. Public Health.

[70] Garzón C., Beveridge B., Gordon M., Martin C., Matalon E., Moore E. (2013). Power, privilege, and the process of community-based participatory research: Critical reflections on forging an empowered partnership for environmental justice in West Oakland, California. Environ. Justice.

[71] Imara N., Lazerow S., Lee A.Y., Malloy N., Orozco A., Pérez C. (2010). East Oakland Particulate Matter 2.5 Community-Based Air Monitoring Research Report.

[72] Brown P., Brody J.G., Morello-Frosch R., Tovar J., Zota A.R., Rudel R.A. (2012). Measuring the success of community science: The Northern California household exposure study. Environ. Health Perspect..

[73] Cohen A., Lopez A., Malloy N., Morello-Frosch R. (2012). Our environment, our health: A community-based participatory environmental health survey in Richmond, California. Health Educ. Behav..

[74] Commodore A., Wilson S., Muhammad O., Svendsen E., Pearce J. (2017). Community-based participatory research for the study of air pollution: A review of motivations, approaches, and outcomes. Environ. Monit. Assess..

[75] Heeks R. (2007). Theorizing ICT4D research. Inf. Technol. Int. Dev..

[76] Karanasios S. (2014). Framing ICT4D research using activity theory: A match between the ICT4D field and theory?. Inf. Technol..

[77] Parmar V. (2009). A multidisciplinary approach to ICT development. Inf. Technol. Int. Dev..

[78] Avgerou C. (2010). Discourses on ICT and Development. Inf. Technol. Int. Dev..

[79] Burrell J., Toyama K. (2009). What constitutes good ICTD research?. Inf. Technol. Int. Dev..

[80] Dearden A. (2012). See No Evil? Ethics in an Interventionist ICTD. Inf. Technol. Int. Dev..

[81] D’Ignazio C., Warren J., Blair D. (2014). The role of small data for governance in the 21st century. Governança Digital.

[82] Brown P. (1997). Popular epidemiology revisited. Curr. Soc..

[83] Brown P. (1992). Popular epidemiology and toxic waste contamination: Lay and professional ways of knowing. J. Health Soc. Behav..

[84] Conrad C.C., Hilchey K.G. (2011). A review of citizen science and community-based environmental monitoring: issues and opportunities. Environ. Monit. Assess..

[85] Den Broeder L., Devilee J., Van Oers H., Schuit A.J., Wagemakers A. (2016). Citizen science for public health. Health Promot. Int..

[86] Pfister D.S., Godana G.D. (2012). Deliberation technology. J. Public Delib..

[87] Kamel Boulos M.N., Resch B., Crowley D.N., Breslin J.G., Sohn G., Burtner R., Pike W.A., Jezierski E., Chuang K.-Y. (2011). Crowdsourcing, citizen sensing and sensor web technologies for public and environmental health surveillance and crisis management: Trends, OGC standards and application examples. Int. J. Health Geogr..

[88] Lupton D. (2015). Health promotion in the digital era: A Critical commentary. Health Promot. Int..

[89] Lupton D. (2014). Beyond techno-utopia: Critical approaches to digital health technologies. Societies.

[90] Lupton D. (2012). M-Health and health promotion: The digital cyborg and surveillance society. Soc. Theor. Health.

[91] NACCHO (2008). YouTube, Facebook, MySpace, Blogs, and More: Innovative Ways Local Health Departments Are Reaching Adolescents.

[92] NACCHO (2009). The Use of Public Health Informatics to Identify and Address Inequities.

[93] Brabham D.C., Ribisl K.M., Kirchner T.R., Bernhardt J.M. (2014). Crowdsourcing applications for public health. Am. J. Prev. Med..

[94] Ozdalga E., Ozdalga A., Ahuja N. (2012). The smartphone in medicine: A review of current and potential use among physicians and students. J. Med. Internet Res..

[95] Patrick K., Griswold W.G., Raab F., Intille S.S. (2008). Health and the mobile phone. Am. J. Prev. Med..

[96] DHHS (2012). Healthy People 2020. http://www.hhs.gov/open/initiatives/mhealth/recommendations.html.

[97] Lemay N.V., Sullivan T., Jumbe B., Perry C.P. (2012). Reaching remote health workers in Malawi: Baseline assessment of a pilot mhealth intervention. J. Health Commun..

[98] Lester R.T., Ritvo P., Mills E.J., Kariri A., Karanja S., Chung M.H., Jack W., Habyarimana J., Sadatsafavi M., Najafzadeh M. (2010). Effects of a mobile phone short message service on antiretroviral treatment adherence in Kenya (WelTel Kenya1): A randomised trial. Lancet.

[99] Redfern J., Thiagalingam A., Jan S., Whittaker R., Hackett M., Mooney J., Keizer L.D., Hillis G., Chow C. (2014). Development of a set of mobile phone text messages designed for prevention of recurrent cardiovascular events. Eur. J. Prev. Cardiol..

[100] Stenner S.P., Johnson K.B., Denny J.C. (2012). PASTE: Patient-centered SMS text tagging in a medication management system. J. Am. Med. Inf. Assoc..

[101] Bravo S., Valero M.A., Pau I. (2012). A Tele-Health communication and information system for underserved children in rural areas of Nicaragua. Inf. Technol..

[102] Tamrat T., Kachnowski S. (2012). Special delivery: An analysis of mhealth in maternal and newborn health programs and their outcomes around the world. Matern. Child Health J..

[103] Brownstein J.S., Freifeld C.C., Madoff L.C. (2009). Digital disease detection—Harnessing the web for public health surveillance. N. Engl. J. Med..

[104] Freifeld C.C., Chunara R., Mekaru S.R., Chan E.H., Kass-Hout T., Ayala Iacucci A., Brownstein J.S. (2010). Participatory epidemiology: Use of mobile phones for community-based health reporting. PLoS Med..

[105] Ranard B.L., Ha Y.P., Meisel Z.F., Asch D.A., Hill S.S., Becker L.B., Seymour A.K., Merchant R.M. (2014). Crowdsourcing—Harnessing the masses to advance health and medicine, a systematic review. J. Gen. Intern. Med..

[106] Chaix B., Kestens Y., Perchoux C., Karusisi N., Merlo J., Labadi K. (2012). An interactive mapping tool to assess individual mobility patterns in neighborhood studies. Am. J. Prev. Med..

[107] Gorely T., Biddle S.J.H., Marshall S.J., Cameron N. (2009). The prevalence of leisure time sedentary behaviour and physical activity in adolescent boys: An ecological momentary assessment approach. Int. J. Pediatr. Obes..

[108] Shiffman S., Stone A.A., Hufford M.R. (2008). Ecological momentary assessment. Annu. Rev. Clin. Psychol..

[109] Zenk S.N., Schulz A.J., Matthews S.A., Odoms-Young A., Wilbur J., Wegrzyn L., Gibbs K., Braunschweig C., Stokes C. (2011). Activity space environment and dietary and physical activity behaviors: A pilot study. Health Place.

[110] WHO (2011). MHealth: New Horizons for Health through Mobile Technologies.

[111] APHA (2012). The New Public Health: Rewiring for the Future.

[112] NACCHO (2012). Local Health Departments and MHealth.

[113] NIH (2014). Office of Behavior and Social Science Research. http://obssr.od.nih.gov/scientific_areas/methodology/mhealth/index.aspx.

[114] CDC (2014). CDC Mobile. http://www.cdc.gov/mobile/index.html.

[115] HRSA (2013). HRSA MHealth. http://www.hrsa.gov/healthit/healthitgranteespotlight/mhealth2013/.

[116] NIH (2014). Wireless Health 2014. http://www.wirelesshealth2014.org/.

[117] Lupton D. (2013). Digitized Health Promotion: Personal Responsibility for Health in the Web 2.0 Era.

[118] Petteway R.J., Mujahid M., Allen A., Morello-Frosch R. (2019). The body language of place: A new method for mapping intergenerational “Geographies of Embodiment” in place-Health research. Soc. Sci. Med..

[119] Petteway R.J. (2019). Intergenerational photovoice perspectives of place and health in public housing: participatory coding, theming, and mapping in/of the “Structure Struggle”. Health Place.

[120] Van Wart S., Tsai K., Parikh T. Local ground: A paper-based toolkit for documenting local geospatial knowledge. Proceedings of the ACM Symposium on Computing for Development (DEV).

[121] Campos-Castillo C. (2015). Revisiting the first-level digital divide in the United States: Gender and race/ethnicity patterns, 2007–2012. Soc. Sci. Comput. Rev..

[122] Chen W., Wellman B. (2004). The global digital divide—Within and between countries. IT Soc..

[123] Tsatsou P. (2011). Digital Divides Revisited: What is new about divides and their research?. Media Cult. Soc..

[124] Borg K., Boulet M., Smith L., Bragge P. (2019). Digital inclusion & health communication: A rapid review of literature. Health Commun..

[125] Bach A., Schaffer G., Wolfson T. (2013). Digital human capital: Developing a framework for understanding the economic impact of digital exclusion in low-income communities. J. Inf. Policy.

[126] Bambra C., Smith K.E., Pearce J. (2019). Scaling up: The politics of health and place. Soc. Sci. Med..

